# The neurobiology of apathy in depression and neurocognitive impairment in older adults: a review of epidemiological, clinical, neuropsychological and biological research

**DOI:** 10.1038/s41398-022-02292-3

**Published:** 2022-12-26

**Authors:** David C. Steffens, Mario Fahed, Kevin J. Manning, Lihong Wang

**Affiliations:** Department of Psychiatry, University of Connecticut School of Medicine, Farmington, CT USA

**Keywords:** Psychiatric disorders, Neuroscience

## Abstract

Apathy is a common condition that involves diminished initiative, diminished interest and diminished emotional expression or responsiveness. It is highly prevalent in the context of a variety of neuropsychiatric disorders and is related to poor health outcomes. Presence of apathy is associated with cognitive and functional decline in dementia. Despite its negative impact on health, there is no definitive treatment for apathy, a clinical reality that may be due in part to lack of knowledge about assessment, neuropsychological features and neurobiological underpinnings. Here, we review and synthesize evidence from clinical, epidemiological, neuropsychological, peripheral biomarker and neuroimaging research. Apathy is a common feature of depression and cognitive disorders and is associated with impairment in executive function. Neuropsychological and neuroimaging studies point to dysfunction of brain circuitry involving the prefrontal cortex, especially the dorsolateral prefrontal cortex circuit, the dorsomedial prefrontal cortex circuit, and the ventromedial prefrontal cortex circuit. However, inconsistent findings, particularly in neuroimaging may be due to heterogeneity of apathy symptoms (with a need to better elucidate subtypes), neuropsychiatric comorbidities, the severity of cognitive impairment and other factors. These factors need to be accounted for in future studies so that biomarker research can make progress. On the whole, the literature on apathy has identified likely neurocognitive, peripheral biomarker and neuroimaging targets for understanding apathy, but also points to the need to address methodological issues that will better inform future studies. In turn, as we learn more about the underpinning of apathy and its subtypes, subsequent research can focus on new neurally based interventions that will strengthen the clinical management of apathy in the context of its comorbidities.

## Introduction

### Apathy: a complex clinical, neurobehavioral and neurobiological construct

Most clinicians caring for older adults recognize that apathy is common in late life, particularly among individuals with depression and dementia. Presence of apathy affects psychiatric and neurological outcomes [1, 2], takes a toll on patients and their families [3], and may herald cognitive and functional decline [4]. Yet many questions about apathy remain regarding its phenomenology, clinical characterization, neuropsychological and neurobiological underpinnings. Is apathy a neuropsychiatric symptom or a behavioral response? Is apathy the same construct in depression as it is in dementia? How does one assess it? How does one conceptualize apathy from a clinical neuroscience perspective? Much of what we know about neural correlates of apathy relates to post-stroke conditions [5], so to what extent does vascular neuropathology play a role in the evolution of apathy?

In this paper, we provide an updated review of the literature on apathy, with a particular focus on its role in depression and neurocognitive impairment, both major (dementia) and minor (syndromes related to mild cognitive impairment). While our goal is to provide a clinical neuroscience-based overview of neurobiological and neuropsychological phenomena related to apathy, we feel it is important to start with a clinical perspective. Given the outstanding questions about presentation of apathy, its characterization and assessment, its impact on a variety of medical and psychiatric conditions, and its treatment, beginning with a review of clinical issues surrounding apathy should provide a strong foundation from which to build a clinical neuroscience understanding of its neurobiological and neurobehavioral basis.

### Apathy: a clinical perspective

Apathy is a common and pervasive neuropsychiatric syndrome [6]. It was originally conceptualized as a “lack of motivation” and has since gained more dimensions as academic endeavors and expert panels have sought to define it [6, 7]. In 2018, apathy was defined as a quantitative reduction of goal-directed activity when compared with a patient’s previous level of functioning. The presentation must persist for at least four weeks and affect at least two of the three apathy dimensions: behavior/cognition; emotion; social interaction [6]. More recently, in 2021, these domains were updated to diminished initiative, diminished interest and diminished emotional expression/responsiveness [7]. Specific diagnostic criteria are shown in Tables 1 and 2.

**Table 1:** Diagnostic Criteria for Apathy and Diagnostic criteria for apathy in neurocognitive disorders [7] (reprinted with permission).

2020 Diagnostic Criteria for Apathy in Neurocognitive Disorders	
Patient needs to meet every criterion below for a diagnosis of Apathy in Neurocognitive Disorders:	
Criterion A: Primary Diagnoses	The patient meets the criteria for a syndrome of cognitive impairment or dementia (as defined by either ICD or DSM-5 criteria; e.g.: AD, vascular dementia, Frontotemporal Dementia, Dementia of Lewy Body, Parkinson Disease Dementia or a pre-dementia cognitive impairment syndrome such as Mild Cognitive Impairment, prodromal Alzheimer’s Disease, or other cognitive disorder).
Criterion B. Symptoms and duration	Symptoms: The patient exhibits at least one symptom in at least two of the following three dimensions (B1 to B3). Duration: These symptoms have been persistent or frequently recurrent for a minimum of 4 weeks and represent a change from the patient’s usual behavior. Note: These changes may be reported by the patient or by observing others.
	Dimension B1: Diminished initiative: Less spontaneous and/or active than usual self: Less likely to initiate usual activities such as hobbies, chores, self-care, conversation, work-related or social activities.
	Dimension B2:Diminished interest: Less enthusiastic about usual activities: - Less interested in, or less curious about events in their environment - Less interested in activities and plans made by others - Less interested in friends and family - Reduced participation in activities even when stimulated - Less persistence in maintaining or completing tasks or activities
	Dimension B3: Diminished emotional expression/responsiveness: - Less spontaneous emotions - Less affectionate compared to their usual self - Expresses less emotion in response to positive or negative events - Less concerned about the impact of their actions on other people - Less empathy
Criterion C: Exclusionary criteria	These symptoms are not exclusively explained by psychiatric illnesses, intellectual disability, physical disabilities, motor disabilities, change in level of consciousness, or the direct physiological effects of a substance.
Criterion D: Severity	These symptoms cause clinically significant impairment in personal, social, occupational, and/or other important areas of functioning. This impairment must be a change from their usual behavior.

**Table 2:** 2018 Consensus panel diagnostic criteria for apathy [6] (reprinted with permission).

2018 Consensus panel diagnostic criteria for Apathy	
CRITERION A:	A quantitative reduction of goal-directed activity either in behavioral, cognitive, emotional or social dimensions in comparison to the patient’s previous level of functioning in these areas. These changes may be reported by the patient himself/herself or by observation of others.
CRITERION B:	The presence of at least 2 of the 3 following dimensions for a period of at least four weeks and present most of the time
	B1. BEHAVIOUR & COGNITION: Loss of, or diminished, goal-directed behaviour or cognitive activity as evidenced by at least one of the following: - General level of activity: the patient has a reduced level of activity either at home or work, makes less effort to initiate or accomplish tasks spontaneously, or needs to be prompted to perform them. - Persistence of activity: He/she is less persistent in maintaining an activity or conversation, finding solutions to problems or thinking of alternative ways to accomplish them if they become difficult. - Making choices: He/she has less interest or takes longer to make choices when different alternatives exist (e.g., selecting TV programs, preparing meals, choosing from a menu, etc.) - Interest in external issue: He/she has less interest in or reacts less to news, either good or bad, or has less interest in doing new things. - Personal wellbeing: He/she is less interested in his/her own health and wellbeing or personal image (general appearance, grooming, clothes, etc.).
	B2. EMOTION Loss of, or diminished, emotion as evidenced by at least one of the following: - Spontaneous emotions: the patient shows less spontaneous (self-generated) emotions regarding their own affairs, or appears less interested in events that should matter to him/her or to people that he/she knows well. - Emotional reactions to environment: He/she expresses less emotional reaction in response to positive or negative events in his/her environment that affect him/her or people he/she knows well (e.g., when things go well or bad, responding to jokes, or events on a TV program or a movie, or when disturbed or prompted to do things he/she would prefer not to do). - Impact on others: He/she is less concerned about the impact of his/her actions or feelings on the people around him/her. - Empathy: He/she shows less empathy to the emotions or feelings of others (e.g., becoming happy or sad when someone is happy or sad, or being moved when others need help). - Verbal or physical expressions: He/she shows less verbal or physical reactions that reveal his/her emotional states.
	B3. SOCIAL INTERACTION Loss of, or diminished engagement in social interaction as evidenced by at least one of the following: - Spontaneous social initiative: the patient takes less initiative in spontaneously proposing social or leisure activities to family or others. - Environmentally stimulated social interaction: He/she participates less, or is less comfortable or more indifferent to social or leisure activities suggested by people around him/her. - Relationship with family members: He/she shows less interest in family members (e.g., to know what is happening to them, to meet them or make arrangements to contact them). - Verbal interaction: He/she is less likely to initiate a conversation, or he/she withdraws soon from it - Homebound: He/she prefer to stays at home more frequently or longer than usual and shows less interest in getting out to meet people.
CRITERION C:	These symptoms (A – B) cause clinically significant impairment in personal, social, occupational, or other important areas of functioning.
CRITERION D:	The symptoms (A – B) are not exclusively explained or due to physical disabilities (e.g. blindness and loss of hearing), to motor disabilities, to a diminished level of consciousness, to the direct physiological effects of a substance (e.g. drug of abuse, medication), or to major changes in the patient’s environment.

#### Epidemiology

Prior studies have shown that the prevalence of apathy in mild cognitive impairment (MCI) ranges between 10.7% and 44.8% [8]. In a meta-analysis by Zhao et al, the most frequent neuropsychiatric symptom of Alzheimer’s Disease (AD) was shown to be apathy, with an overall prevalence of 49% (95% CI 41–57%) [9]. Apathy is also the most persistent neuropsychiatric symptom in AD patients [10]. In the behavioral variant of Frontotemporal Dementia (FTD), the prevalence of apathy is staggering, with ranges of 62 to 89% [11]. The prevalence of apathy in Dementia with Lewy Bodies ranges from 35 to 100% [12]. As a potential harbinger of dementia, apathy was linked to an approximately 2-fold increased risk of incident dementia in patients presenting to memory clinics [13]. Once dementia starts, apathy is associated with worsening functional decline, higher caregiver burden, early institutionalization with increased cost of care resulting, and increased mortality [14].

#### Clinical assessment

Whether considered a symptom or a standalone disorder, apathy has been the subject of much debate. Across the literature, many terms have been used to describe similar presentations to apathy: amotivation, avolition, abulia, withdrawal, social isolation, etc. To delineate a symptomatic apathy syndrome, consensus statements have been issued by expert groups and are detailed in Tables 1 and 2 (6, 7). Both sets of criteria call for the exclusion of other psychiatric disorders. This is especially difficult with depression, as it may have a similar presentation to apathy, especially in older adults. Both apathy and depression present with decreased interest, decreased initiative, decreased motivation, impaired concentration and libido, and reported fatigue. They are, however, distinct but often co-occurring syndromes [15, 16]. Brodaty and Connors suggested the following features to differentiate apathy and depression: In apathy, the lack of emotion and indifference, passivity, lack of rumination and anxiety, and increasing severity over the course of dementia set the presentation apart from depression wherein the patient would present dysphoric, hopeless, avoidant of treatment and socialization, comorbid with changes in sleep, appetite, possibly suicidal, and with a course that would fluctuate with medication trials and treatment [17]. In depression, an individual may still find social relationships to be valuable despite an absent drive to pursue them while a person with apathy no longer finds worth in social relationships [18].

To reliably assess apathy and track therapeutic interventions, use of validated scales is generally recommended, especially the Apathy Evaluation Scale and the Dementia Apathy Interview and Rating. The Apathy Evaluation Scale (AES) [19] measures apathy regardless of etiology or diagnosis and has been validated for use in a variety of clinical populations, including nursing home residents. It is sensitive to change over four weeks and capable of tracking apathy in three domains (cognitive, behavioral, and emotional). Importantly, its semi-structured interview format exists for all three information sources: patients themselves, caregivers and clinicians [19]. The Dementia Apathy Interview and Rating (DAIR) gathers information in a semi-structured interview conducted by the clinician with a patient or an informant, and is capable of tracking changes at four weeks, which is especially useful for monitoring outcomes of interventions for patients with Alzheimer’s Disease [20].

Other promising avenues look to technology to gather data that can supplement and inform clinical information such as passive environmental sensing technology.

#### Treatment

There are no established clinical guidelines for treatment of apathy [15]. With regards to nonpharmacological interventions, a systematic review found that therapeutic interventions such as stimulation, creative activities, Montessori methods, cooking, and individually tailored behavioral interventions yield positive outcomes. There was not a high level of evidence for music therapy, exercise groups, multisensory stimulation, or pet therapy [21]. “Engage,” a behavioral therapy aimed at reward exposure, was found to be effective for depression [22].

There are no FDA-approved medications for apathy. The utility of selective serotonin reuptake inhibitors (SSRIs) for apathy in depression is unclear. The literature on the topic includes several case reports that described onset or worsening of apathy with SSRIs in depression [16]. In one study, methylphenidate was not effective in enhancing SSRI treatment of apathy in depression [23]. Another study found that while bupropion was safe, it was not efficacious compared with placebo in the treatment of apathy in patients with Alzheimer’s dementia in the absence of clinically relevant depressed mood [24]. In Lewy Body Dementia, acetylcholinesterase inhibitors (AchEIs) were found to be useful in a systematic review of four studies [12]. For FTD, Hermann et al. found citalopram to be associated with a decrease in apathy on the Frontal Behavioral Inventory [25]. In a preliminary study, agomelatine, an antidepressant with MT1 and MT2 receptor agonism and 5-HT2C receptor antagonism, decreased apathy scores on the AES – clinician version (AES-C) in patients with FTD as compared to melatonin. The medication was well-tolerated and was also correlated with decrease in caregiver distress [26]. Looking to the future, a phase II trial is currently underway for the use of intranasal oxytocin for behavior in FTD [27].

For treatment studies of apathy in AD, we consider the ADMET trial. In this six-week, randomized, phase II, double-blind, placebo-controlled multicenter trial enrolling Alzheimer’s disease participants with apathy assigned to methylphenidate 20 mg daily or placebo, there was a statistically significant reduction in CGI-C and Neuropsychiatric Inventory apathy scores with active treatment, but there was no treatment difference on the AES [28]. A Phase III clinical trial, ADMET II, is currently underway [29]. In another study, AES-C scores were significantly improved (after adjusting for baseline apathy) with a trial of methylphenidate up to 20 mg daily in a 12-week, double-blind, randomized, placebo-controlled trial in a cohort of older community-dwelling veterans [30]. A 2018 meta-analysis demonstrated further support for methylphenidate as a treatment for apathy [31]. Though some studies show slight improvement of apathy with cholinesterase inhibitors, the aforementioned meta-analysis did not find high-quality evidence to support the use of any cholinesterase inhibitor (donepezil, galantamine or rivastigmine) for apathy in Alzheimer’s [31]. There is no evidence to support the discontinuation of cholinesterase inhibitors as an intervention to decrease apathy either [24, 30]. Low-quality studies showed slight improvement of apathy with antipsychotic and antidepressant use in Alzheimer’s disease [31].

Repetitive transcranial magnetic stimulation (rTMS) was shown to improve AES-C scores significantly compared with sham treatment in a randomized, double-blind, parallel-arm, sham-controlled pilot study in subjects presenting with apathy in AD. The authors proposed several mechanisms for this finding, including an enhanced dopamine transmission, increased neuronal activity in prefrontal regions, and neurotrophic/neuroprotective effects when the DLPFC (dorsolateral prefrontal cortex) is stimulated. This effect was significant at four weeks but not maintained at 8- or 12-week follow-up, possibly due to a small sample of 20 participants and short duration of treatment [32].

### Apathy: a perspective linking neuropsychology and brain circuitry

The core definition of the apathy syndrome is a reduction in motivated goal-directed behavior. Engaging in motivated behavior requires a cost-benefit evaluation of whether the reward associated with that potential behavior outweighs the cost of performance [33]. Motivation to obtain rewards is clearly decreased in major depression [34]. Depressed patients are less willing to exert effort to obtain rewards and apathy appears to further reduce the incentive of rewarding outcomes [35, 36]. Motivated behavior also requires the engagement of executive functions – or the cognitive control mechanisms of response inhibition, working memory, and mental flexibility that sustain attention to the task at hand, engage monitoring and evaluation of task progress, and enable the ability to subsequently change direction if need be [37]. The behavioral syndrome of apathy is therefore thought to originate in brain regions responsible for both executive functioning and reward/motivation. For example, three recent reviews provide insight into imaging findings related to apathy in AD, pointing to frontostriatal circuit involvement, including the anterior cingulate cortex (ACC), prefrontal cortex (PFC) and parts of the basal ganglia, particularly the ventral striatum, including the nucleus accumbens and olfactory tubercle [38–40].

Three frontostriatal circuits underlie executive functioning and motivational states. These circuits include the dorsolateral prefrontal cortex (DLPFC) circuit, the dorsomedial prefrontal cortex (DMPFC), and the ventromedial prefrontal cortex (VMPFC) circuit. All three circuits originate in the prefrontal cortex and have direct and indirect pathways that project to the striatum, then to the globus pallidus and substantia nigra, and onto to the thalamus before looping back to the prefrontal cortex [41]. The three circuits run in close proximity to one another in parallel through shared common brain structures and receive inputs from neurotransmitter systems that help modulate behavior. Dopaminergic systems – in particular the mesolimbic and mesocortical pathways – assist in the regulation of motivational behavior and executive functioning [42]. The mesolimbic pathway begins in the ventral tegmental area and projects to the nucleus accumbens, limbic system, and the medial prefrontal cortex, while the mesocortical pathway connects the ventral tegmental area to the prefrontal cortex [43]. Overall, then, frontostriatal circuitry works in conjunction with dopaminergic pathways to allow for the effective pursuit of motivated behaviors [44].

#### Circuitry underlying executive functioning and apathy

The DLPFC circuit is closely associated with the core executive functions of working memory, response inhibition, and mental flexibility which are foundational processes for sustained attention, planning, and problem solving [37]. Clinically, disruption of the DLPFC circuit results in susceptibility to distraction, poor multi-tasking, organizational difficulties, and concrete or rigid thinking [45]. On neuropsychological examination, patients with lesions to the DLPFC often demonstrate difficulty with tasks such as the Wisconsin Card Sorting Test (requiring mental flexibility and self-monitoring) and the Stroop Color Word Interference Task or other “Go/No-go” tests requiring sustained attention and response inhibition [41]. Poor performance on the Wisconsin Card Sorting Test, Stroop Task, and other tests of executive functioning are commonly observed in older adults with major depression. Apathy may at least partially explain the association between executive dysfunction and depression in older adults. Funes et al. [46] found a significant association between depression severity and performance on the Stroop and Trail Making Test in LLD patients, but the strength of this association was no longer significant after apathy was entered into the statistical model. Several lines of evidence suggest that dopaminergic dysfunction contributes to the connection between apathy and cognitive impairment in LLD although direct evidence is lacking. First, animal models reveal depletion of dopamine in the DLPFC and medial prefrontal cortex results in impairments in attention and working memory and this deficit can be subsequently reversed with dopamine agonists [47]. Preliminary evidence with human subjects supports this finding. Rutherford et al. found a dose-dependent increase of L-DOPA on processing speed tasks in 36 older adults with depression. Finally, dopamine (D_2/3_) receptor availability in the nucleus accumbens is negatively correlated with apathy measured with the AES in depressed adults [48].

The DMPFC circuit originates in the anterior cingulate cortex (ACC), a prominent hub of both cognitive and reward/emotional processing. The ACC influences behavior through the initiation and monitoring of action – guiding attention in response to changes in motivation and incentive [41]. Structural changes to the ACC are therefore associated with depression and apathy [16, 49], and at the most extreme, lesions in this region result in akinetic mutism where patients have intact consciousness but are seemingly indifferent to sensory stimulation, thirst, or hunger [41]. On testing, lesions to the ACC result in significant deficits in cognitive tasks that require initiation and generation (e.g., fluency tasks) [50]. From the ACC, the DMPFC circuit projects to the ventral striatum, which includes the ventromedial caudate, ventral putamen, as well as key structures involved in reward (nucleus accumbens) and olfaction (olfactory tubercle). These connections may explain why reward systems are often found to be disrupted in apathetic patients (see more below) and why apathy is correlated with olfactory dysfunction [51].

The vmPFC circuit originates in the lateral orbital gyrus of Brodmann’s area 11 and the medial inferior frontal gyrus of the areas 10 and 47 [41]. Damage to the VMPFC results in difficulty integrating the motivational, emotional, and social aspects of behavior into decision-making. Clinically, disruption of the VMPFC results in both emotional blunting and mood lability, poor judgement, as well as lack of social tact. Profound apathy has also been reported following rare bilateral lesions to basal ganglia-VMPFC circuitry with a reduction of apathy following treatment with dopamine agonists [52]. On cognitive exam, perseverative errors – or the inability to disengage from a previous pattern of responding despite a change in stimulus – have been found to be associated with decreased volume in the VMPFC in older adults with depression [53].

VMPFC circuitry is also engaged by effort or reward-based decision-making tasks – such as the Effort Expenditure for Rewards Task (EEfRT) and Iowa Gambling Task (IGT). During the IGT participants choose cards from one of four decks with the goal to win as much money as possible [54]. Selections from two of the IGT decks are associated with higher immediate reward yet greater long-term loss, whereas the other two decks have lower immediate but better long-term gain. In order to achieve success, participants must use gain/loss feedback to guide decision-making and identify the advantageous decks. The IGT has also been used with older adults with major depression. McGovern and colleagues found older adults with depression and apathy performed in an advantageous manner on the IGT and selected more cards from the conservative decks compared with non-apathetic depressed older adults [55]. In this instance, IGT performance appears to capture a failure to become incentivized or respond to rewards in older apathetic depressed adults.

#### Apathy is independent from executive functioning

Apathy and executive dysfunction commonly travel together in older adults. For example, an early study by McPherson et al. found that AD patients with apathy performed significantly worse compared with AD patients without apathy on several measures of executive functioning including response inhibition, cognitive flexibility, and sustained attention [56]. Yet, it is also worth noting that several studies have found performance on tests of executive function to be independent of apathy. Marin and colleagues found no association between behavioral markers of apathy and executive functioning tests (including verbal fluency) and tests of global cognition in 52 older adults with major depression and cognitive impairment [57]. Brodaty noted that change scores in apathy were not related to subsequent change scores in executive functioning [58]. Thus, in some cases, the presence of apathy may be solely related to abnormalities in the DMPFC and not associated with classic executive functioning tests representative of DLPFC functioning [59].

#### Association between apathy and dementia risk in MCI

Increases in the prevalence of apathy coincide with increases in dementia severity and frontostriatal degeneration in neurodegenerative diseases such as AD, Parkinson’s disease, FTD and Huntington’s disease. For example, the estimated prevalence of apathy is 18% in MCI or very mild AD patients and 39 and 48% in mild and moderate AD patients [60] (see van Dyck et al. for an excellent review of the neurobiology of apathy in AD [61]). The presence of apathy has been therefore suggested as an early behavioral marker of impending dementia. An early study of 131 patients with amnestic MCI followed upwards of four years found that patients with both amnestic-MCI and a clinical diagnosis of apathy at baseline had an almost sevenfold risk of progression to AD compared to amnestic-MCI patients without apathy after controlling for age, sex, education, baseline global cognitive and functional status, and depression [62]. Subsequent larger studies have revealed similar findings. Using a sample of 1,821 participants with amnestic and non-amnestic MCI from the National Alzheimer’s Coordinating Center (NACC) database, Rosenberg et al. found that the presence of apathy, irritability, and elation were the only neuropsychiatric symptoms independently associated with a subsequent transition to AD after controlling for demographics and baseline cognitive and functional status [63]. Likewise, a subsequent study also using NACC data compared the risk of developing AD in MCI patients without neuropsychiatric symptoms to MCI patients with apathy, depression, or comorbid apathy and depression [64]. Of note, MCI patients with both apathy and depression had the greatest risk of developing AD. Those with apathy only also had an elevated risk, but not those with depression only.

#### Association between apathy and risk of cognitive decline in community-dwelling older adults

Apathy has also been linked to a risk of cognitive decline and dementia in community-dwelling older adults without clear evidence of MCI at baseline. Clarke et al. investigated the association between apathy and cognitive decline in 1,136 older adults who participated in the Baltimore Epidemiologic Catchment Area (ECA) study [65]. Efforts were made to exclude participants with significant cognitive impairment (based upon a Mini-Mental State Exam or MMSE of less than 24) and depression at baseline. Twenty-three percent of the sample endorsed the presence of apathy at baseline using self-report. Analyses revealed an interesting association between apathy, cognition, and function. While greater baseline apathy was associated with cognitive decline (defined as a three-point difference on the MMSE) at one year after controlling for demographics and follow-up depression, change in apathy from baseline to one-year was not associated with one-year cognitive change. Apathy likewise was not associated with cognitive decline in the long-term (year 13), although apathy was associated with greater functional decline at year 13, which is consistent with evidence that apathy explains considerable variance in older adults’ everyday functioning even when accounting for depression and cognition [66, 67].

### Apathy: a neurobiological perspective incorporating peripheral biomarkers and neuroimaging

#### Peripheral biomarkers and apathy

Across a variety of neurological and neuropsychiatric conditions, there is a growing literature on peripheral biomarkers related to presence and severity of apathy. In particular, there appear to be links between apathy and inflammatory markers. Among AD patients, compared with those without apathy, those with apathy had higher levels of interleukin-6, interleukin-10, and leptin [68]. In a study combining two large national cohorts (National Alzheimer’s Coordinating Center, NACC, *n* = 22,760 and Alzheimer’s Disease Neuroimaging Initiative, ADNI, *n* = 1733), lower Aβ_42_ was associated with a steep increase in apathy, while higher tau was associated with a marked decrease in apathy over a five-year period [69]. Among a cohort of stroke patients, post-stroke apathy at six months was significantly associated with elevated C-Reactive Protein (CRP) concentrations [70]. In a principal component analysis (PCA) study of patients with early Parkinson’s Disease (PD), compared with healthy control subjects, the PC with elevated IL-2 and IL-6 was associated with faster progression of Non-Motor Symptoms Scale total and mood/apathy domain scores [71].

There are also negative studies in this area. In the Leiden Study of adults 85 years and older, no association at baseline was found between C-reactive protein (CRP) concentration and apathy or depression [72]. In subjects free of apathy and depression at baseline, subjects in the highest CRP-tertile at baseline had significantly more increase in depressive symptoms but not in apathy symptoms during follow-up. While the authors concluded that apathy and depression in old age may have different etiologies, it is important to note that no inference on causality can be established. In a study of persons with HIV infection ages 50 and older, the presence of apathy was not associated with any component of a panel of potential biomarkers, including tumor necrosis factor-alpha, kynurenine, tryptophan, quinolinic acid, brain-derived neurotrophic factor, glial fibrillary acidic protein, neurofilament light chain, and phosphorylated tau at position threonine 181 [73].

#### Neuroimaging and apathy: an overview

As indicated in the prior section, and consistent with prior reviews on the topic, apathy has largely been attributed to structural and functional changes in the frontostriatal circuitry, particularly the ACC, OFC, ventral striatum, and insula. From a network perspective then, apathy has most often been tied to dysfunction in reward and salience networks, although increasing evidence suggests an additional role for the Executive Control Network (and its primary hub, the DLPC) in the etiology of apathy [74–76]. However, neuroimaging research has also yielded inconsistent findings, which are thought to be attributable to a variety of methodological issues. It is thought that such discrepancies in findings may be due to differences in apathy subtypes (i.e., variability in which symptoms predominate; see multidimensional model below), the severity of comorbid cognitive impairment, type of neuroimaging modality used in the study, imaging outcome measures, and other confounding factors (e.g., gender, race/ethnicity, education, and comorbid personality traits). These challenges result in difficulties in the validation of neuroimaging markers and have limited the clinical application of neuroimaging in the assessment of apathy. Below, we expand upon recent reviews of apathy and neuroimaging by focusing on multi-modal studies as well as investigations into apathy sub-constructs. In Table 3, we list relevant studies of apathy in AD by imaging modality. We also note similarities and differences in the neural substrates of apathy among neurodegenerative disorders and late-life depression and end with a discussion on the correlation between apathy and ischemic changes. Fortunately, with the advancement of artificial intelligence and other data analyses techniques, as well as the rapid growth of large imaging database worldwide, it is believed that neuroimaging will not only be utilized to provide information on neural mechanisms, but also can become a clinical tool assisting differential diagnoses, monitoring clinical progression and treatment effects, and development of new therapeutic targets.

**Table 3:** Neuroimaging studies of apathy in Alzheimer’s disease (AD).

Authors & year	Imaging modality	Apathy scale	Study sample	Study findings
Craig et al., [96]	99m Tc-HMPAO SPECT	NPI	Probable AD (*n* = 31)	Apathy was associated with more severe hypoperfusion in the prefrontal and anterior temporal cortex
Ott et al., [97]	99m Tc-HMPAO SPECT	AES	Possible AD (*n* = 40)	Lower right temporoparietal perfusion was correlated with apathy
Benoit et al., 1999 [98]	99m Tc-ECD SPECT	NPI	AD (*n* = 20)	Hypoperfusion of the anterior cingulate cortex (ACC)
Benoit et al., 2002 [98]	99m Tc-ECD SPECT (SPM99)	NPI	Apathetic AD (*n* = 15), non-apathetic AD (*n* = 15), healthy controls-HC (*n* = 11)	Decreased perfusion of left ACC, right inferior and medial gyrus frontalis, left orbitofrontal cortex (OFC) and right gyrus lingualis was found in the apathetic subgroup compared with the non-apathetic AD subgroup
Benoit et al., [81]	99m Tc-ECD SPECT	Apathy Inventory	AD (*n* = 30)	Apathy was associated with lower brain perfusion in bilateral superior OFC, and to a lesser extent in left middle frontal gyrus (BA10). Lack of initiative score was correlated with hypoperfusion in right ACC. Lack of interest score correlated with hypoperfusion in right middle OFC. Emotional blunting score correlated with hypoperfusion in left superior dorsolateral prefrontal cortex (dlPFC)
Tanaka et al., [99]	SPECT	NPI	Mild to moderate AD (*n* = 70) treated with donepezil for 12 weeks	Donepezil responder (n = 21), non-responder (n = 42), worsened (n = 7). Apathy and depression in AD patients involved distinct functional circuits. Dysphoria, anxiety and apathy significantly improved among the responder group. CBF in the premotor and parietotemporal cortices was higher in the responder group than in the worsened group.
Robert et al., [82]	SPECT	Apathy Inventory	AD *(n* = 30)	Lack of initiative and lack of interest were associated with hypoperfusion in right ACC
Lanctôt et al., [100]	SPECT and sMRI	NPI	Apathetic AD (*n* = 23) non- apathetic AD (*n* = 23) healthy controls (*n* = 23)	The AD patients with lack of initiative and interest showed a significantly lower perfusion in the ACC than the AD patients without lack of initiative and interest.
Kang et al., [101]	99m Tc-HMPAO SPECT	NPI	AD (*n* = 81)	Apathetic non-depressed patients had lower regional perfusion in the right amygdala, temporal, posterior cingulate (PCC), right superior frontal, postcentral, and left superior temporal gyri than non-apathetic patients. Apathy and depression in AD patients involved distinct functional circuits.
Oka et al., [102]	99m Tc-HMPAO SPECT	NPI-Q and Dysexecutive Questionnaire score (DEX)	AD (*n* = 27)	Lower baseline regional cerebral blood flow in several frontal areas, including the dlPFC and ventrolateral prefrontal cortex (vlPFC), the ACC, and the OFC predicted greater reductions in the score for apathy and DEX after patients switched from donepezil to galantamine therapy
Mega et al., [103]	[18F]FDG-PET	NPI	Mild to moderate AD (*n* = 19) galantamine treatment	Right ACC glucose metabolism increase significantly correlated with improvement in depression, and right ventral putamen metabolic increase correlated with improvement in apathy
Holthoff et al., [104]	[18F]FDG-PET	NPI	AD with apathy (*n* = 17), AD without apathy (*n* = 36)	Hypometabolism of left OFC in the apathetic AD group relative to the non-apathetic group
Marshall et al., [105]	[18F]FDG-PET	Scale for the Assessment of Negative Symptoms in AD (SANS-AD)	AD with apathy (*n* = 14), AD without apathy *n* = 27)	Reduced glucose metabolism in ACC, medial orbitofrontal (mPFC) region and bilateral medial thalamus were found in the apathetic group
Schroeter et al., [106]	[18F]FDG-PET	NPI	54 subjects mainly with early AD, frontotemporal lobar degeneration, and subjective cognitive impairment	Apathy was associated with hypometabolism in the ventral tegmental area (VTA), a component of the motivational dopaminergic network
Gatchel, et al. [107]	[18F]FDG-PET ROI analysis	NPI-Q	CN (*n* = 104) amnestic MCI (*n* = 203) mild AD (*n* = 95) ADNI data	Cross-sectionally, the PCC hypometabolism was correlated with higher apathy scores. In longitudinal analysis, baseline PCC hypometabolism was associated with higher apathy over time and baseline supramarginal gyrus hypometabolism was positively associated with rate of change in apathy over time
Delrieu et al. [108]	MRI (volume) [18F]FDG-PET	NPI	MCI with apathy (*n* = 11), MCI without apathy (*n* = 54)	Decreased metabolism of PCC
Ballarini et al. [109]	[18F]FDG-PET Voxel-wise interregional correlation analysis	Neuropsychiatric symptoms (NPSs) sub-syndromes (SSy)	early-age-of-onset AD (*n* = 51), among which 27 had NPSs data, HC (*n* = 57)	The apathetic subsydrome score was negatively correlated with metabolism in the bilateral OFC and dlPFC
Marshall et al. [78]	PIB-PET (amyloid) FDG-PET (glucose uptake) ACC, OFC, precuneus, SMA	AES	MCI (*N* = 24)	No association between apathy and regional FDG metabolism, but a significant association between increased apathy and greater cortical PiB retention independent of age
Mori et al. [77]	PIB PET (amyloid) ROI analysis	NPI	AD with PIB + (*n* = 28)	Apathy severity was associated with greater PIB retention in bilateral frontal and right ACC. Presence of apathy was associated with greater PIB retention in bilateral frontal cortex
Kitamura et al. [79]	(11)C-PBB3-PET for tau (11)C-PIB-PET for Aβ sMRI dMRI	Apathy Scale (AS)	AD with high AS (*n* = 10) AD with low AS *(n* = 7)	Elevated (11)C-PBB3 SUVR in OFC, decreased OFC thickness and decreased FA in the uncinate fasciculus (UNC) correlated with AS. Path analysis indicated that increased (11)C-PBB3 SUVR in OFC affects apathy directly and through reduction of OFC thickness and subsequent decrease in fractional anisotropy of UNC.
Johansson et al. [110]	(18)F-flutemetamol-PET sMRI, FreeSurfer T2 FLAIR for white matter lesions (WML), LST toolbox	AES	CN (*n* = 104), MCI (*n* = 53) followed for up to 4 years	Apathy and anxiety were shown related to Aβ deposition and predicted cognitive decline. Apathy level, but not anxiety, was associated with atrophy in the ACC, PCC, lateral temporal, and parietal cortex, as well as white matter lesion volume
Sun et al. [111]	Aβ PET, ROI - medial orbitofrontal cortex (mOFC) and the pars orbitalis cortex (POFC).	NPI	non-demented apathy(+) *n* = 114, among which 78 with Aβ PET, non-demented apathy (-) *n* = 943, among which 569 with Aβ PET, 5–6 year follow-up (ADNI data)	Individuals with apathy, higher CSF Aβ42 level, or frontal lobe Aβ deposition had an increased risk of cognitive decline compared with those without apathy. Subjects with higher frontal Aβ deposition had a higher risk for apathy conversion to cognitive decline
Lavretsky et al., [112]	sMRI	Psychiatric Evaluation section of the Minimum Uniform Dataset (MUDS) of the California Alzheimer’s Disease Centers Program	270 community-dwelling elderly cognitively intact (38%), cognitively impaired (27%), demented (35%)	Apathy was associated with higher total volume of lacunes in the white matter
Bruen et al., [113]	sMRI (voxel-based morphometry,VBM)	NPI	Mild AD (*n* = 31)	Apathy was associated with gray matter density loss in the ACC and frontal cortex bilaterally, the head of the left caudate nucleus, and bilateral putamen
Tunnard et al., [114]	sMRI	NPI	Mild to moderate AD (*n* = 111)	Apathetic patients had significantly greater cortical thinning in left caudal ACC, left lateral OFC, and left superior and vlPFC regions compared with those without apathy
Kim et al., [115]	sMRI dMRI	NPI	Very mild or mild probable AD (*n* = 51)	Apathy group showed significantly lower fractional anisotropy (FA) values than apathy-free group in the left anterior cingulum (A-C). Left A-C FA values were negatively correlated with apathy severity
Ota et al., [116]	sMRI dMRI	Apathy Scale	Probable AD (n = 21)	Apathy was associated with lower white matter integrity index (FA) in the ACC and medial thalamus.
Tighe et al., [117]	sMRI dMRI	NPI	MCI (*n* = 22), AD (*n* = 23)	Participants with the lowest anterior cingulum (A-C) fractional anisotropy tertile were more likely to exhibit irritability, agitation, dysphoria, apathy, and nighttime behavioral disturbances compared to those in the highest tertile. However, only irritability remained significant after adjusting for MMSE.
Stanton et al. [118]	sMRI, VBM	Apathy defined by Robert criteria	AD (*n* = 17), progressive supranuclear palsy (*n* = 17)	Apathy was associated with atrophy of the vmPFC, OFC, and left insula in both AD and PSP. Reduced initiative associated with atrophy of the ACC and vlPFC. Emotional blunting was assocated with atrophy of the left insula.
Donovan et al. [119]	sMRI, (cortical thickness) CSF Aβ1–42	NPI-Q change over time	CN (*n* = 229) MCI (*n* = 395) AD (*n* = 88)	Reduced baseline inferior temporal cortical thickness was predictive of increasing apathy over time, and reduced supramarginal cortical thickness was predictive of increasing hallucinations over time
Moon et al. [120]	sMRI	NPI-Apathy	AD (*N* = 40)	Apathy and irritability associated with decreased volume of bilateral ACC and right posterior insula
Guercio et al. [121]	sMRI (cortical thickness)	AES-C	MCI (*n* = 47), HC (*n* = 19)	Lower inferior temporal cortical thickness was predictive of greater apathy. Greater ACC cortical thickness was also predictive of greater apathy
Zahodne, et al. [89]	sMRI	NPI	MCI (*n* = 334)	Depression was associated with reduced cortical thickness in the entorhinal cortex at baseline and accelerated atrophy in the ACC. Apathy did not correlate with atrophy
Huey et al. [122]	sMRI FreeSurfer whole-brain ROI volumes VBM	NPI	mild AD (*n* = 57)	Atrophy of the following regions were independently associated with apathy: the ventromedial prefrontal cortex (vmPFC), ventrolateral prefrontal cortex (vlPFC); PCC and adjacent lateral cortex; and the bank of the superior temporal sulcus.
Kumfor et al. [123]	sMRI	NPI-Q	AD (*n* = 53)	Affective apathy was associated with the vPFC, behavioral apathy with the basal ganglia, and cognitive apathy with the dmPFC. Finally, affective and behavioral apathy significantly predicted carer burden
Garcia-Alberca, et al. [124]	sMRI & FLAIR Scheltens visual rating scale	NPI	Probable AD (*n* = 46)	Increased total medial temporal atrophy (MTA) was significantly associated with apathy. WMH measures did not significantly predict any BPSD item
Wei et al. [85]	sMRI	Dimensional Apathy Scale (DAS)	AD-early (*n* = 10), AD-late (*n* = 10), Behavioral variant-FTD (bvFTD)-early (*n* = 22), bvFTD-late (*n* = 22) HC (*n* = 28)	In the early stage of the disease (< 5 years since onset), emotional apathy was greater in bvFTD than AD. In contrast, in the late stage (> 5 years since onset), executive apathy was greater in AD than bvFTD, although apathy was elevated across all dimensions compared to controls.
Chan et al., [125]	sMRI FreeSurfer ROI analysis	NPI	Cognitive impairment (CI) With Apathy (*n* = 96), CI Without Apathy (*n* = 311)	Cortical GM was thinner in the right mOFC and left rACC and thicker in the left MTC in CI participants with apathy relative to CI participants without apathy.
Nour et al. [126] }	sMRI FreeSurfer ROI analysis (ADNI data)	NPI	HC (*n* = 35), AD with anxiety (*n* = 27), AD with depression (*n* = 19), AD with apathy (*n* = 24)	The left insula had a strong negative association with Clinical Dementia Rate Sum of Boxes and AD Assessment Scale-cognitive subscale-13 items in anxiety and apathy groups. The difference in GM density in the left insula and hippocampus plays a crucial role in depression, anxiety, and apathy
Chaudhary, et al. [127]	sMRI		Meta-analysis control *(n* = 59), AD *(n* = 167), Validation MCI/AD (*n* = 19), HC (*n* = 25)	Label-based review showed atrophy in the ACC, putamen, insula, inferior frontal gyrus (IFG) and middle temporal gyrus (MTG) in AD patients with apathy. Right putamen and MTG showed gray matter volume was in negative correlation with AES, behavioral, and emotional scores, and right IFG with emotional score. Putamen, MTG and IFG atrophy in AD-associated apathy, potentially independent of CI and depression
Johansson, et al. [128]	sMRI, T2FLAIR CSF amyloid-beta [Aβ]42/Aβ40 ratio, plasma phosphorylated tau	AES	Cognitively unimpaired older adults (*n* = 356) followed up for 8 years	Aβ pathology at baseline was associated with increasing levels of apathy longitudinally. More rapid decline of cognition over time was related to increasing levels of apathy
Moon et al. [93]	FLAIR for WMH Hypertension and cardiovascular events, Diabetes mellitus and hyperlipidemia	NPI	AD (*n* = 162)	Hypertension was correlated with the severity of apathy. The remaining vascular factors were not significant. Presence of hypertension and asymptomatic stroke are related with the severity of apathy and depression in Alzheimer’s dementia.
Sarabia-Cobo et al. [94]	WMH		AD (*n* = 109 MCI (*n* = 59)	The older group with AD had a higher prevalence of leukoaraiosis and apathy, with significant differences compared to the MCI group
Torso et al. [129]	sMRI FLAIR for WML voxel-lesion-symptom mapping (VLSM)	NPI	Amnestic MCI (*n* = 31, 32% at 2-year follow-up, HC (*n* = 29)	VLSM revealed a strict association between the presence of lesions in the anterior thalamic radiations (ATRs) and the severity of apathy. Regional grey matter atrophy did not account for any BPSD.
Misquitta, et al. [92]	sMRI FLAIR for WMH	NPS	AD (*n* = 121), MCI (*n* = 315), HC *(n* = 225)	The focal grey matter atrophy and WMH volume both contributed significantly to NPS subsyndromes in MCI and AD subjects, and WMH burden played a greater role.
Hahn, et al. [130]	sMRI, TBSS	NPI and IA	Apathetic AD (*n* = 30), Non-apathetic AD (*n* = 30)	The apathy group had significantly reduced FA values in the genu of the corpus callosum compared to the non-apathy group. The severity of apathy was negatively correlated with FA values of the left anterior and posterior cingulum, right superior longitudinal fasciculus, splenium, body and genu of the corpus callosum and bilateral uncinate fasciculus in the apathy group
Tokuchi, et al. [131]	sMRI FLAIR for WML	NPI	subcortical ischemic vascular disease (SIVD, *n* = 24) AD (*n* = 32)	MD value within the right superior longitudinal fasciculus and CDR predicted the apathy domain. Distinct patterns of regression models between SIVD and AD including the left superior longitudinal fasciculus to the hyperactivity domain, the left uncinate fasciculus/forceps major to the psychosis domain, and the right superior longitudinal fasciculus to the apathy domain.
Setiadi, et al. [132]	dMRI (DTI)	AES-C	aMCI (*n* = 29), HC (*n* = 20)	In aMCI, higher severity of apathy was associated with lower FA in various white matter pathways including the left anterior part of inferior fronto-occipital fasciculus/uncinate fasciculus, genu and body of the corpus callosum, superior and anterior corona radiata, anterior thalamic radiation of both hemispheres and in the right superior longitudinal fasciculus/anterior segment of arcuate fasciculus
Balthazar, et al. [133]	rsfMRI networks: default-mode (DMN) salience (SN) frontoparietal control (FPCN), attention (AN)	NPI	mild to moderate AD *(n* = 20), HC (*n* = 17)	There was a significant association between greater affective factor symptoms and reduced FPCN connectivity. There was no association between the hyperactivity factor and any of the networks. There was an association between greater apathy and reduced FPCN connectivity
Jones, et al. [134]}	rsfMRI networks: default-mode (DMN) salience (SN) frontoparietal control (FPCN), attention (AN)	NPI	Apathetic AD (*n* = 35), non-apathetic AD (*n* = 35)	Apathetic patients had reduced connectivity between the left insula and right superior parietal cortex. Apathetic patients also had increased connectivity between the right dlPFC seed and the right superior parietal cortex.
Tumati, et al. [135]	rsfMRI functional connectome graph theory	NPS-A factor analysis on NPS: Affective factor Hyperactivity factor	Apathy AD (*n* = 21), non-apathy non-NPS AD (*n* = 28) non-apathy with other NPS AD (*n* = 38)	Apathy was associated with increased participation coefficient in the frontoparietal and cingulo-opercular template-based networks. AD patients showed higher modularity compared to controls at the whole brain level and higher participation coefficient in the ventral attention network. Loss of segregation in the frontoparietal and cingulo-opercular network, which are involved in the control of goal-directed behavior, was associated with apathy in MCI/AD
Amanzio, et al., [136]	tfMRI go/no-go task	Awareness of Deficit Questionnaire-Dementia scale.	Unaware AD (*n* = 14), Aware AD *(n* = 15)	Unaware patients showed reduced activation in the right post-central gyrus, the associative cortical areas such as the right parieto-temporal-occipital junction and the left temporal gyrus, the striatum, and the cerebellum.
Aguera-Ortiz, et al. [137]	sMRI,dMRI, T2 FLAIR	Apathy in Dementia-Nursing Home Version Scale	Moderate to severe nursing home AD (*n* = 37)	Bilateral damage to the corpus callosum and internal capsule was associated with apathy severity. A smaller and more anteriorly located region of the right internal capsule and corpus callosum was associated with higher emotional blunting
Raimo, et al. [138]	meta-analysis PET, SPECT, MRI	Apathy	PD 15 studies; AD 15 studies; FDG-PET 14 studies; FTD 13 studies; [11c]PET 2 studies, SPECT 6 studies. fMRI 4 studies	Overall analysis showed that apathy is associated with hypometabolism and a decreased gray matter volume in the left IFG. an altered brain perfusion and decreased gray matter volume in ACC (BA 24, 32) in AD, decreased gray matter volume in IFG (BA 44, 45) and parietal cortex (BA 40) in FTD patients.
Alexopoulos, et. al, [88]	fMRI ROI-to-ROI FC Nacc and ACC as ROIs	AES	HC (*n* = 10), LLD with apathy (*n* = 7), LLD without apathy (*n* = 9)	Apathetic depressed patients had lower FC of the NAcc with emotion/reward related regions (amygdala, caudate, putamen, globus pallidus) and thalamus, lower FC of dACC with dorsal executive related regions (the superior, middle, and inferior frontal gyruses, right superior parietal and VLPFC), increased FC of the NAcc with dorsomedial PFC (dACC and dmPFC), and higher FC within salience network (dACC-insula) and FC of the dACC with OFC and dlPFC.
Yuen, et al., [139]	rsfMRI ROI-to-voxel FC right anterior insula, rAI as ROIs	AES	HC (*n* = 10), LLD with apathy (*n* = 7) LLD without apathy (*n* = 9)	LLD with apathy had lower FC within salience network (rAI-dACC) and higher rAI-dlPFC and rAI-PCC FC.
Oberlin, et al., [140]	rsfMRI, dMRI ROI-to-Voxel functional connectivity and structrual connectivity	AES	LLD with apathy (*n* = 20) LLD without apathy *(n* = 20) Among the 40 LLD, 12-week nonrandomized single-group trial of escitalopram, 27 remitted	Relative to nonapathetic depression, those depression with apathy had lower FC of SN seeds with the dlPFC, premotor cortex, mid CC, and paracentral lobule and greater FC of SN with the lateral temporal cortex and temporal pole. Compared with depression without apathy, those with apathy had lower structural connectivity in the splenium, cingulum, and fronto-occipital fasciculus.
Onoda, et al., [90]	rsfMRI, graph theory	Apathy Scale (AS) Self-rating Depression Scale (SDS)	middle-aged and older adults (*n* = 392). mild depression (*n* = 79) apathy (*n* = 66) both depression & apathy (*n* = 33)	ACC showed lower nodal efficiency, local efficiency, and betweenness centrality in apathy, whereas in depression, it showed higher nodal efficiency and betweenness centrality. The ACC constitutes “salience network (SN), which detects salient experiences. These results indicate that apathy is characterized by decreased salience-related processing in ACC, whereas depression is characterized by increased salience-related processing.

*ADNI* Alzheimer’s Disease Neuroimaging Initiative, *AES* Apathy Evaluation Scale, *CBF* cerebral blood flow, *CN* cognitively normal, *FDG-PET* fluorodeoxyglucose-positron emission tomography, *HC* healthy control, *MCI* mild cognitive impairment, *NPI* Neuropsychiatric Inventory, *NPS* neuropsychiatric symptoms, *PIB* Pittsburgh compound B, *rsfMRI* resting-state functional MRI, *SPECT* single photon emission computed tomography.

#### Multi-modality neuroimaging in apathetic AD

Neuroimaging studies on the neural correlates of apathy in AD have covered a wide range of neuroimaging techniques including T1 structural magnetic resonance imaging (sMRI) for brain structural volume and/or cortical thickness, T2 fluid-attenuated inversion recovery (FLAIR) for white matter lesions/hyperintensities (WML/WMH), diffusion tensor imaging (DTI, or dMRI) for measures of fractional anisotropy (FA), mean diffusivity (MD) or tractography, fluorodeoxyglucose-positron emission tomography (FDG-PET) for metabolism or perfusion, Pittsburgh compound B (PiB)-PET for Aβ deposition, (11)C-PBB3-PET for tau imaging, SPECT for blood flow and resting-state fMRI (rsfMRI) and task-related fMRI (tfMRI). As demonstrated in Table 3, converging evidence suggests a disruption of the frontal-striatal circuit in apathetic AD patients. Across these studies, apathy in AD showed a significant correlation with hypoperfusion of the anterior cingulate cortex (ACC), orbitofrontal cortex (OFC), ventromedial prefrontal cortex (vmPFC), putamen, and posterior cingulate (PCC) in SPECT and FDG-PET studies; cortical thinning and lower volume in these regions in sMRI studies; and lower FA in the corpus collosum, longitudinal fasciculus, and uncinate fasciculus in dMRI studies. Apathy was also correlated with greater Aβ depositions in the ACC, PFC, and putamen [77, 78] but not in one study [79], and with high tau accumulation in the OFC [79].

In addition to 11C-PBB3 PET scans for tau deposits, Kitamura and colleagues [79] also conducted PE 11C-PiB PET scans to examine Aβ deposition in AD patients with high (*n* = 10) and low (*n* = 7) Apathy Scale scores. They also acquired T1-weighted imaging to measure regional cortical thickness and diffusion tensor imaging to measure FA for white matter integrity. They investigated the association among focal Aβ and tau deposits, neural loss of focal brain, disruption of connected fibers and the severity of apathy. While they did not find significant differences in Aβ deposition between AD patients with high vs. low apathy scores, they did find an association between apathy and elevated tau deposition in OFC, decreased OFC cortical thickness, and decreased fractional anisotropy (FA) in the uncinate fasciculus, which connects to the OFC. Further path analysis indicated that the increased tau accumulation in OFC could affect apathy directly and through the reduction of OFC thickness and subsequent decrease of FA in uncinate fasciculus. This is one of the very few studies that has taken advantage of multi-modality neuroimaging data and pursued the causal relationship among findings from multi-modality imaging data. However, studies on PET often suffer from a small sample size. Even without PET imaging to confirm the pathology, more studies using multi-modality neuroimaging are still needed to better understand the disrupted brain regions or neural networks related to apathy.

#### A multidimensional model of apathy and associated neural circuits

**Fig. 1:**
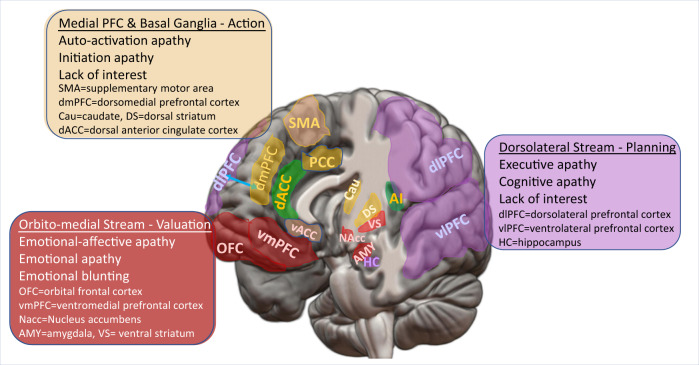
Multimodality model of apathy (following Levy and Dubois [76]). Brain regions of the same color contribute to the same subtype of apathy. The dorsal anterior cingulate cortex (dACC) and the right anterior insula (AI) comprise the salience network (SN), which monitors switches between internal and external attention and facilitates motivation and decision-making. The dACC connects to the ventral, dorsal, and medial-motor networks, and dysfunction of the dACC can contribute to each subtype of apathy.

Apathy is not a unitary construct. Levy and Dubois proposed three subtypes: emotional-affective, cognitive, and auto-activation apathy [80] to form a multidimensional model (see Fig. 1). *Emotional apathy* (also known as emotional-affective apathy), which is characterized by emotional blunting and reduced empathy, is related to the disruption of *the orbito-medial stream* (OFC, vmPFC, or ventral striatum). *Executive apathy* (also known as cognitive apathy) is characterized by difficulties in planning and organizing goals for the future and is related to the disruption of the dorsolateral stream (dlPFC, vlPFC, frontal pole, and dorsal caudate). *Initiation apathy* (also known as auto-activation apathy), characterized by indolence or lack of initiation, is related to deficits in the basal ganglia, the dmPFC, the premotor medial PFC, and ventral ACC. Inconsistency in these reported imaging results may be due in part to variability of different apathy subdomains across studies. The majority of studies on apathy used the Neuropsychiatric Inventory (NPI), which does not have multidimensional constructs for studying the subdomain-associated neural mechanisms.

Only a few studies examined subdomain-related neural substrates [81, 82]. Benoit and colleagues used the Apathy Inventory (AI) to measure the three types of apathy and found that “emotional blunting” was correlated with hypoperfusion in left superior dlPFC, “lack of interest” was correlated with hypoperfusion in right middle OFC, and “lack of initiative” was correlated with hypoperfusion in right ACC. The results do not support the hypotheses of Levy and Dubois. Using the three subdomain scores, Robert and colleagues subgrouped AD patients into “with lack of initiative and lack of interest” (*n* = 19) and “without lack of initiative and interest” (*n* = 12) [82]. When comparing AD patients with and without “lack of initiative and interest” and controlling for “emotional blunting” and NPI depression scores, the subgroup with lack of initiative and interest showed significantly lower perfusion in the right ACC than the subgroup without lack of initiative and interest. Therefore, ACC hypoperfusion is associated with “lack of initiative”, “lack of initiative and interest” as well as the total apathy score. This is not surprising given that ACC is considered a dorsal nexus [83, 84] that connects to several neural networks and plays an important role in both cognitive and emotional processing. The ACC, together with the right insula, comprise the salience network (SN), which connects with the executive control network (ECN) and default mode network (DMN) and serves as an attention switch between internally focused and externally focused mode. The ACC is also a key hub that links the valuation function of the ventromedial prefrontal cortex (vmPFC) and the orbitofrontal cortex (OFC) to the decision-making and action-taking functions of the dlPFC and supplementary motor cortex, so as to guide attention, monitor error and conflict, and modify motivation and incentive [41]. It is important to note that dysfunction of ACC is not specific to apathy; rather, it can also be found in a number of psychiatric symptoms such as depression, anxiety, and irritability.

Another SMRI study examined gray-matter intensity in AD and behavioral-variant frontotemporal dementia (bvFTD) [85]. Regardless of dementia subtype, emotional apathy was associated with loss of grey matter intensity in the cerebellum, vmPFC, and the amygdala. Executive apathy was associated with reduced integrity of the dlPFC and OFC regions. Initiation apathy was associated with lower grey matter intensity in the mPFC and ACC. These findings are concordant with the neurocircuits hypothesized in the model of multidimensional model of apathy [80].

#### Commonality and differences of neural substrates of apathy among neurodegenerative disorders

Apathy commonly occurs in neurodegenerative disorders such as Parkinson’s disease (PD), AD, frontotemporal dementia (FTD), and Huntington’s disease (HD). A natural area of inquiry is whether apathy of these neurodegeneration disorders involves the same neural circuits and to what extent there is overlap in brain circuitry across disorders. Based on the limited number of existing studies that have sought to address this question, the presence of apathy associated with each neurodegenerative disorder appears to be linked to more predominant neural changes. Go and colleagues compared apathy and brain atrophy between frontotemporal dementia (bvFTD, n = 30) and AD (n = 18) patients [86]. They used the Frontal System Behavior Scale (FrSBe) to assess apathy and rated bran atrophy level for the orbital (OFC), medial (mPFC), dorsolateral (dlPFC) and total prefrontal cortices (PFC) using a 5-point Likert scale ranging from 0 to 4. Patients with bvFTD showed higher incidence of behavioral disturbances than AD with apathy being the most significant. BvFTD patients also demonstrated the highest incidence of atrophy in the medial and orbital frontal cortex and this atrophy was correlated with apathy. Wei and colleagues also compared apathy and brain atrophy between bvFTD and AD, using the Dimensional Apathy Scale (DAS) to quantify the emotional, executive, and initiation dimensions of apathy. They also subgrouped bvFTD and AD each into two group based on the disease duration (time since onset of first symptoms) divided into either “early” (<5 years) or “late” stages (>5 years). Voxel-based morphometry (VBM) was used to investigate differences in grey matter intensities between groups. In the early stage of the disease (< 5 years since onset), emotional apathy was present in bvFTD but not in AD. In contrast, executive apathy was more severe in the late stage (>5 years since onset) of AD compared with bvFTD. These findings can inform the development of appropriate treatment targets to ameliorate the impact of apathy in dementia, though future research is still needed to determine the extent of overlap of neural findings related to apathy across various neuropsychiatric conditions.

#### Comparisons of neural substrates of apathy between AD and depression

Similar to comparisons on neural substrates of apathy among neurodegenerative diseases, there are very few studies compared the similarity and differences in neural mechanisms of apathy between AD and late-life depression. Although there are no studies that directly compared apathy-related neuroimaging features between AD and late-life depression (LLD), one systematic review [87] compared findings of neuroimaging studies on apathy in LLD, brain injury, AD and other neurodegenerative disorders. The limited studies of apathy in LLD showed altered functional connectivity (FC) within the reward network and SN, with increased FC of the Nucleus Accumbens (NAcc) with dmPFC) and higher FC within the SN [88]. In contrast, Yuen and colleagues found that apathetic LLD patients had decreased (not increased) FC in the SN [16].

Apathy and depression also have overlapping components. Depression is often associated with dysfunction in the SN and reward-related network, so it follows that studies are needed that directly compare depression- and apathy-associated neural network function. A few studies directly compared the neural correlates between depression and apathy [89, 90]. Onoda et al. examined graph properties of the functional connectome in older adults with depression (*n* = 79), with apathy (*n* = 66), or with both depression and apathy (n = 33) [90]. The ACC showed lower nodal efficiency and betweenness centrality in apathy, while it showed higher nodal efficiency and betweenness centrality in depression. The results indicate that salience-related processing in the ACC is decreased in apathy and increased in depression, supporting the notion that apathy and depression are distinctive constructs.

#### Contribution of cerebrovascular factors to apathy in AD

Another understudied issue related to apathy in AD is whether apathy is due to AD pathology or due to microvascular structural damages and cerebrovascular disease. Studies using T2 FLAIR to examine white matter lesions (WML) or white matter hyperintensities (WMH) have partly addressed this question [91–94]. One large study (*n* = 651) found that focal grey matter atrophy and WMH burden were associated with worsening neuropsychiatric symptoms (NPS) including apathy over time in MCI and AD, with WMH having the greater contribution to NPS [92]. Increased number of vascular risk factors worsened affective NPS. This and other studies examining WMH [87–90] are generally in agreement that worsening cerebrovascular disease contributes to the presence of apathy in AD.

#### Other methodological issues in current neuroimaging studies in apathy

Many neuroimaging studies have used small sample sizes and did not control for general levels of disease severity. This holds true particularly for PET and SPECT studies but was also the case for early sMRI studies as well. In these small-sample studies, one should be concerned about embracing any notions of causality. In addition, prespecified region-of-interest (ROI) analyses often predominate rather than atheoretical whole-brain studies. One caution with such “hypothesis-driven” approaches is creation of a bias in the literature that could lead to the perception of a well-established consistent association between apathy and one or more specific regions. More multi-modality studies using state-of-art data analyses methods will reduce this hypotheses-driven induced bias in the literature.

## Conclusions and future directions

Apathy is a common and complex condition that clinically occurs alone but more frequently in the context of a variety of neuropsychiatric disorders. It is often found accompanied by executive dysfunction. While there are no FDA-approved pharmacological treatments for apathy, there is a mixed literature with some support for use of methylphenidate, SSRIs and cholinesterase inhibitors for apathy when it occurs in certain conditions. Repetitive TMS may hold promise as a treatment. The underlying pathophysiology is linked to pro-inflammatory processes that affect key regions of the brain, especially the frontostriatal circuits. Neuroimaging studies have helped identify possible biomarkers of apathy, but inconsistent findings may result from heterogeneity of expression of apathy (i.e., variability in which symptoms predominate), presence of comorbid neuropsychiatric disorders in which apathy is common, a spectrum of severity of cognitive function, and a variety of sociodemographic factors.

Future research should focus on identifying underlying processes and brain circuitry related to apathy across a variety of conditions, especially in depression and Alzheimer’s disease and related dementias. Such studies will help elucidate the heterogeneity of apathy and point to more tailored treatments. As potential candidate interventions are identified, subsequent clinical trials will need to account for confounding factors such as mood and cognition, and the presence of psychomotor slowing or parkinsonism [95].
